# Priming of Plant Resistance to Heat Stress and Tomato Yellow Leaf Curl Thailand Virus With Plant-Derived Materials

**DOI:** 10.3389/fpls.2019.00906

**Published:** 2019-07-12

**Authors:** Wei-An Tsai, Sung-Hsia Weng, Ming-Cheng Chen, Jeng-Shane Lin, Wen-Shih Tsai

**Affiliations:** ^1^Hualien District Agricultural Research and Extension Station, Council of Agriculture, Executive Yuan, Hualien City, Taiwan; ^2^Queensland Alliance for Agriculture and Food Innovation, The University of Queensland, St Lucia, QLD, Australia; ^3^School of Biological Sciences, The University of Queensland, St Lucia, QLD, Australia; ^4^Department of Life Sciences, National Chung Hsing University, Taichung, Taiwan; ^5^Department of Plant Medicine, National Chiayi University, Chiayi City, Taiwan

**Keywords:** anise oil, eugenol, jasmonic acid, salicylic acid, thermotolerance, tomato (*Solanum lycopersicum*), tomato yellow leaf curl Thailand virus (TYLCTHV)

## Abstract

Plants are often simultaneously exposed to diverse environmental stresses, and can tune suitable responses to them through hormones. Salicylic acid (SA) and jasmonic acid (JA) signaling pathways are known to enhance resistance against heat stress and tomato yellow leaf curl Thailand virus (TYLCTHV) infection. However, there is limited information regarding alternative natural priming agents against heat stress and viruses. In this study, two plant-derived priming agents, eugenol and anise oil, were tested for their roles in conferring thermotolerance and virus resistance in tomato plants. Under heat stress, the survival rates and average fresh weight were higher in plants treated with eugenol or anise oil than in control plants. These two priming agents were further tested for antiviral activities. After TYLCTHV infection, the disease incidence and relative abundance of TYLCTHV were lower in anise oil- and eugenol-treated plants than in control plants. Further analyses revealed that a few SA, JA, and RNA silencing genes were enhanced in the former. Moreover, SA, JA, and H_2_O_2_ contents increased considerably after eugenol and anise oil treatments. Our findings imply that anise oil and eugenol initiated SA- and JA-mediated defenses to promote thermotolerance and antiviral responses of tomato plants.

## Introduction

Plants are often exposed to multiple biotic and abiotic stresses throughout their lifespan. Their survival requires sophisticated sensory systems that perceive various stress signals and activate the most appropriate responses at the molecular, cellular, and physiological levels.

Under natural conditions, certain stresses that occur sequentially or simultaneously can enhance plant tolerance to other abiotic or biotic stresses (Foyer et al., 2016; Hossain et al., 2018). Several common signaling components, including ROS, plant hormones, proline, and stress-responsive genes, are triggered to initiate a cross-tolerance mechanism in response to environmental stresses (Yasuda et al., 2008; Islam et al., 2009; Atkinson and Urwin, 2012; Lin et al., 2014). Interestingly, the stimuli that induce plants to enter a primed state are the preceding stresses as well as natural or synthetic chemical compounds (Savvides et al., 2016). The naturally occurring metabolic stimuli include vitamins, hormones, amino acids, growth-promoting molecules, and reactive oxygen-nitrogen-sulfur species that initiate complex signal transduction pathways and alter gene expression to enhance plant tolerance to abiotic and biotic stresses (Athar et al., 2008; Arnao and Hernandez-Ruiz, 2014; Christou et al., 2014; Li T. et al., 2014; Savvides et al., 2016). In wheat, ascorbate decreases oxidative damage and maintains ion homeostasis under salt stress conditions (Athar et al., 2008). In *Arabidopsis thaliana*, auxin response factors were shown to influence heat tolerance through regulating plant development and HSP expression (Lin et al., 2018). Additionally, in higher plants, SA and MeJA not only improve heat stress tolerance (Snyman and Cronje, 2008; Clarke et al., 2009; Wang et al., 2010), they also enhance antiviral activities (Calil and Fontes, 2017; Li et al., 2017, 2018). A previous study revealed that a proline treatment of tobacco cells results in increased antioxidant enzyme activities to mediate cadmium stress tolerance (Islam et al., 2009). Other studies proved that melatonin regulates plant growth as well as antioxidative responses in plants (Arnao and Hernandez-Ruiz, 2014, 2018; Zhang et al., 2015), whereas beta-aminobutyric acid promotes biotic and abiotic stress tolerance (Ton et al., 2009; Jisha and Puthur, 2016; Meller et al., 2018). Eugenol (4-allyl-2-methoxyphenol) elevates nitric oxide and SA accumulation to mediate resistance to TYLCV in tomato plants (Wang and Fan, 2014; Sun et al., 2016). In addition to naturally occurring stimuli, benzothiadiazole and kresoxim-methyl have been synthesized and applied to induce plant defenses (Filippou et al., 2016).

Plant-derived materials have been used to protect plants from many diseases. For example, cinnamon oil, fennel oil, origanum oil, and thyme oil exhibit antifungal activities against *Fusarium* species (Park et al., 2017), whereas lemon oil can inhibit the growth of three phytopathogenic fungi (*Eutypa* sp., *Botryosphaeria dothidea*, and *Fomitiporia mediterranea*) that infect grapevine wood (Ammad et al., 2018). In addition to their direct antimicrobial effects, plant-derived materials have also been reported to synergistically and directly activate or prime treated plants to promote responses to biotic and abiotic stresses (Chandrashekhara et al., 2010; Ertani et al., 2013, 2014; Rouphael et al., 2017). The essential oil from *Gaultheria procumbens* induces SA-mediated defense responses and resistance to *Colletotrichum higginsianum* in *A. thaliana* (Vergnes et al., 2014). *Nicotiana glutinosa* treated with *Melaleuca alternifolia* (tea tree) oil significantly inhibits lesion development following exposure to TMV (Bishop, 1995). Furthermore, the organic matrix hydrolyte from alfalfa (*Medicago sativa* L.) increases plant biomass under saline conditions (Ertani et al., 2013).

Global warming is one of the major threats to sustainable agriculture. It is responsible for yield losses and increases the severity of adverse effects of abiotic and biotic stresses during vegetable production. The genus *Solanum* includes important and diverse vegetable crop species that are grown worldwide. These crops are highly susceptible to the effects of high temperatures, which may disrupt fruit set and cause cellular injuries. Under heat stress conditions, plants activate heat tolerance mechanisms. For example, HSPs are produced to facilitate the refolding of damaged proteins (Wang et al., 2004). Additionally, the defense hormone SA helps mediate the heat stress tolerance of diverse crops (Larkindale et al., 2005; Wang et al., 2010). Exogenous SA reportedly enhances the antioxidant system and promotes APX and glutathione reductase activities to modulate *Vitis vinifera* thermotolerance (Wang and Li, 2006; Khan et al., 2013). Moreover, the priming of *Solanum lycopersicum* and *Cucumis sativus* with SA alleviates damage of heat stress through upregulating *HSP70* and HSF expression levels (Snyman and Cronje, 2008) or increasing the maximum quantum yield of photosystem II (Shi et al., 2006).

Elevated temperatures interfere with plant physiological processes, and can also inhibit the resistance to pathogenic viruses. A previous study confirmed that high temperatures suppress the TSWV-mediated HR of *Capsicum annuum* (Moury et al., 1998). Another study revealed high temperatures decrease the abundance of the N protein responsible for resistance of *Nicotiana tabacum* to TMV (Zhu et al., 2010). Additionally, viral infections decrease plant tolerance to high temperatures, as suggested by the downregulated transcription and translation of heat-inducible genes in tomato plants infected by TYLCV (Anfoka et al., 2016).

Tomato yellow curl viruses are the most devastating viral pathogens infecting tomato plants (Varma and Malathi, 2003). TYLCTHV, is a bipartite begomovirus (family *Geminiviridae*), mainly transmitted by *Bemisia tabaci* biotype B, and it has been the predominant tomato-infecting begomovirus in Taiwan since 2007 (Tsai et al., 2011; Weng et al., 2015). In tomato plants, the overexpression of *Solanum lycopersicum mitogen-activated protein kinase* 3 (*SlMPK3*) upregulates the expression of SA/JA-mediated defense-related genes (e.g., *pathogenesis-related gene 1* (*PR1*), *PR1b*/ *leucine aminopeptidase A* (*SlLapA*), *proteinase inhibitor I-I* (*SlPI-I*), and *SlPI-II*) to enhance their resistance to TYLCV (Li et al., 2017). The application of exogenous SA and MeJA on tomato leaves can significantly induce *SlMPK3* expression to mediate defense response mechanisms (Yi et al., 2015). Moreover, SA and JA inhibit viral replication as well as cell-to-cell and long-distance viral movements (Shang et al., 2011; Faoro and Gozzo, 2015).

Both SA-mediated defense and RNA silencing are involved in resistance to TYLCV (Li et al., 2018). RNA silencing is an important antiviral mechanism in plants (Burgyan and Havelda, 2011; Calil and Fontes, 2017; Mäkinen et al., 2017). Long double-stranded RNAs, the triggers of RNA silencing, are processed by Dicer-like (DCL) proteins generating vsiRNAs, the guide strand of which is loaded into Argonaute-containing silencing complexes to initiate specific viral RNA degradation (Hedil and Kormelink, 2016). *SlDCL2*/*SlDCL4*-silenced tomato plants show reduced resistance to TYLCV (Li et al., 2018). AGO2 mediates RNA silencing antiviral defenses against the potato viruses potato virus X (Jaubert et al., 2011) and bamboo mosaic virus (Alazem et al., 2017).

Hormone-based defenses against heat stress and viruses have been observed in diverse plant species. However, there is some controversy regarding the strategy of applying synthetic hormones (e.g., SA) to protect plants from various stresses. Although synthetic SA enhances plant defense responses against stresses, it is also detrimental for plant development (Sun et al., 2015). As an alternative, an environmentally friendly antiviral agent, eugenol, was evaluated to increase the resistance of tomato plants to TYLCV (Wang and Fan, 2014; Sun et al., 2016). The application of eugenol increased SA contents and upregulated *SlPer1* expression (Sun et al., 2016). Unfortunately, there is a lack of information regarding the development of alternative natural priming agents effective against heat stress and viruses. To the best of our knowledge, different plant species have similar defense mechanisms for mitigating the effects of abiotic and biotic stresses. In this study, we evaluated heat tolerance of tomato plants treated with natural priming agents, and subsequently the resistance of these tomato plants to TYLCTHV was assessed. The agent-induced defense mechanism in tomato plants was also studied.

## Materials and Methods

### Application of Natural and Synthetic Chemicals

Eugenol was purchased from Sigma-Aldrich (St. Louis, MO, United States). Anise oil was derived from a commercial product (Taiwan Tekho Fine-Chem, Co., Ltd.). The final reagent concentration and treatment of plants were based on a previously described procedure (Wang and Fan, 2014). The abaxial surface of tomato leaves were evenly sprayed with 200 μg mL^–1^ eugenol or anise oil which was prepared in 0.05% Tween 80 (control solution) 24 h before inoculation with TYLCTHV.

### Plant Materials

Tomato (*Solanum lycopersicum* L.) cultivar ‘Know-You 301’ was used in this study. Seeds were surface-sterilized with 0.5% NaClO for 5 min and then washed with distilled water. Seeds were germinated and grown in a greenhouse at 25°C with a 12-h light/12-h dark photoperiod. Tomato seedlings approximately 10 cm tall with 3–5 true leaves (around 14 days after sowing) were used for thermotolerance and virus transmission assays. Seedlings at the 4-true-leaf stage were exposed to viruliferous whiteflies for TYLCTHV inoculation in order to investigate virus infection and accumulation. Each experiment was completed with at least three independent biological replicates.

### Ion Leakage and Phenotypic Analysis in Thermotolerance Assays

The heat tolerance of tomato plants was determined based on the seedling survival rate, average fresh weight, and the results of an ion leakage assay. Specifically, 12-day-old tomato seedlings were individually sprayed with a control solution, anise oil and eugenol three times per day. At 8 h after the last spray treatment, seedlings were exposed to heat stress at 45°C for 12 h. The survival rate and average fresh weight were recorded 5 days later. To evaluate membrane stability, the harvested leaves were subjected to a leakage assay 3 days after heat treatment, which was conducted according to a modified version of a previously described method (Camejo et al., 2005). Leaves were collected and washed three times with de-ionized water to eliminate any external residues. Samples were placed in glass tubes with 20 ml of de-ionized water and left in the dark at 25°C for 20 h. Then, the conductivity values (C1) of aqueous solutions were examined immediately with a conductivity meter (SUNTEX SC-170, Suntex Instruments, Co., Ltd., Taipei, Taiwan) Subsequently, the sample was autoclaved at 121°C for 30 min in order to kill the tissues and then the conductivity (C2) of this solution was recorded. The percentage of electrolytes was calculated as follows: electrolyte (%) = C1/C2 × 100.

### TYLCTHV Infection

Tomato yellow leaf curl Thailand virus isolate LY5 (GenBank Accession No. GU723742) was provided by The World Vegetable Center (Tainan, Taiwan), and was maintained on tomato cultivar ‘ANT22’ via whitefly-mediated transmission. *Bemisia tabaci* B biotype laboratory colonies (with *mtCO1* sequences that are identical to GenBank Accession No. EU4 27726) were derived from individuals collected from poinsettia (*Euphorbia pulcherrima*) plants grown in fields in Hualien City, Taiwan. The colonies were maintained on Chinese kale (*Brassica oleracea*) in whitefly-proof cages at 28°C with a 14-h light/10-h dark photoperiod. For each whitefly biotype, colony purity was monitored every five generations with a PCR assay using biotype-specific primers (Ko et al., 2007). Whiteflies, especially the *B. tabaci* B biotype, transmit TYLCTHV with an efficiency of 80% after an 8-h AAP (Weng et al., 2015). In the current study, adult whiteflies were provided a 48-h AAP. The abaxial surfaces of tomato leaves were evenly sprayed with 200 μg mL^–1^ eugenol or anise oil 24 h before two potentially viruliferous whiteflies were transferred to each tomato seedling. The whiteflies were enclosed in a small net bag (6 cm × 15 cm, 110 mesh) with a leaf for a 48-h IAP (Supplementary Figure S1A). Fifteen replicates were inoculated. After the IAP, the whiteflies were removed from the plants, which were then treated with a systemic insecticide (acetamiprid, 1:1500 dilution) to kill any remaining insects.

### Virus Identification and Quantification in Tomato Plants

To analyze the relationship between TYLCTHV DNA abundance and plant susceptibility, the disease incidence and TYLCTHV genomic DNA content were analyzed. The 2^nd^ leaves of seedlings at the four-true-leaf stage were exposed to viruliferous whiteflies for 48 h of IAP. Then, the 3^rd^ leaves were collected 0, 5, 10, and 15 days after the IAP for DNA extraction analysis from three seedlings (Supplementary Figure S1A). A PCR assay was used to assess TYLCTHV infection of plants at 15 days after the 48-h IAP. The TYLCTHV-specific primer set [THAV3 (5′-CCACATCGTCTTYGTTCTG-3′) and THAC3 (5′-CTTAAYYTTRATATTYTCATCCATCCA-3′)] was used and a previously described PCR protocol (Rojas et al., 1993). The expected size of the amplified product was 1,516 bp. The relative TYLCTHV DNA-A (GU723742.1) and DNA-B (GU723754.1) expression levels were determined by quantitative PCR. The qPCR assay was done with the Power SYBR Green Master Mix (Life Technologies, Carlsbad, CA, United States), gene-specific primer sets (Supplementary Table S1), and the QuantStudio 3 system (Life Technologies). The qPCR program was as follows: 95°C for 5 min; 40 cycles of 95°C for 10 s, 58°C for 15 s, and 72°C for 20 s. The annealing temperature was modified depending on the melting temperature of each primer set, and each reaction was repeated at least three times. GAPDH was used as internal controls for calculating relative DNA amounts. Relative TYLCTHV DNA contents were normalized against tomato plants at 0 dpi. The qPCR data were analyzed using the 2^–Δ⁢Δ⁢*Ct*^ method.

### Gene Expression Analysis

The effects of anise oil and eugenol on the transcript levels of defense-related genes in tomato were examined by RT-qPCR. The following genes were analyzed: TYLCTHV genes, *AC1* and *BC1*; heat-related genes, *heat stress transcription factor A2* (*SlHSFA2*), *SlHSFB1*, *heat shock protein101* (*SlHSP101*), *SlHSP90*, and *SlHSP17.6*; antioxidants genes, *ascorbate peroxidase 2* (*SlAPX2*) and *catalase 2* (*SlCAT2*); SA-related defense genes, *SlPR1*, *SlPR1b*, *non expressor of PR1* (*SlNPR1*), *alternative oxidase 1a* (*SlAOX1a*), and *SlAOX1c*; RNA silencing genes, *SlDCL2*, *SlDCL4*, *SlAGO1A*, *SlAGO1B*, *SlAGO2A*, *SlAGO2B*, *SlRDR1*, and *Ty-1*; and JA-related defense genes, *SlPI-II*, *polyphenol oxidase* (*SlPPO*), *lipoxygenase D* (*SlLoxD*), *SlMAPK3*, and *coronatine-insensitive 1* (*SlCOI1*). RT-qPCR primer sets are listed in Supplementary Table S2. Experiments were conducted in a growth chamber set at 25°C with a 16-h light/8-h dark photoperiod and 80% relative humidity. Plant samples were collected at 0, 8, and 24 h after the treatments with anise oil, eugenol, or control solution. Total RNA was extracted with TRIzol Reagent (Thermo Fisher Scientific, Waltham, MA, United States) according to the manufacturer’s instructions. The RNA was then reverse transcribed with M-MLV Reverse Transcriptase (Thermo Fisher Scientific), and the resulting cDNA was amplified by qPCR using primer sets shown in Supplementary Table S2. The qPCR program was as follows: 95°C for 5 min; 40 cycles of 95°C for 10 s, 60°C for 15 s, and 72°C for 20 s. The annealing temperature was modified depending on the melting temperature of each primer set, and each reaction was repeated at least three times. The *β-actin* (NM_001330119.1) expression level was used as an internal control. Relative gene expression levels were normalized against the gene expression levels in untreated plants or plants treated with control solution. Data were analyzed with the 2^–Δ⁢Δ⁢*Ct*^ method.

### Determination of Salicylic Acid and Jasmonic Acid Contents

The total SA and JA contents in tomato leaves were determined with the Plant SA ELISA kit (#MBS9314138) and Plant JA ELISA kit (#MBS9315634) (MyBioSource, San Diego, CA, United States), respectively. Harvested leaf tissues (approximately 0.1 g) were ground to a fine powder in liquid nitrogen and then mixed with 1 mL PBS (10 mM phosphate buffer, pH 7.4, 150 mM NaCl). Samples were centrifuged (1,000 × *g* for 20 min at 25°C), after which a 50-μL aliquot of the supernatant was loaded into the wells of a 96-well plate (MyBioSource, San Diego, CA, United States). To quantify the SA content, wells were prepared with one of six standard concentrations (ranging from 0.5 to 16 μg mL^–1^). To quantify the JA content, wells were prepared with one of six standard concentrations (ranging from 0.25 to 8 nM). Other than the different standard concentrations, the SA and JA contents were measured using the same procedure. After adding the horseradish peroxidase-conjugated reagent to each well, the plate was incubated at 37°C for 1 h, after which each well was washed four times with washing buffer. The optical density (450 nm) of each well was measured with an ELISA reader within 15 min after adding Stop Solution. Relative SA and JA levels were normalized against those levels in untreated tomato plants.

### Determination of Reactive Oxygen Species Content by Histochemical Staining

H_2_O_2_ content in tomato leaves was determined with 3,3-diaminobenzidine (DAB, Sigma-Aldrich) as a substrate (Orozco-Cardenas and Ryan, 1999). Each detection was done in triplicate with 10 seedling plants for each repeat. The upper leaves of seedlings at the four-true-leaf stage were excised and treated with 1 mg/mL DAB solution (pH 3.8) via an incision. The treated leaves were incubated at 25°C for 20 h in dark. The leaves were then decolorized in a boiling solution of 3:1:1 ethanol: lactic acid: glycerol. After cooling, leaves were submerged in the same solution and photographed under a light microscope. In addition, H_2_O_2_ content was also quantified by titanium chloride method as described by Jana and Choudhuri (1982).

### Data Analysis

All data were analyzed with Student’s *t*-test in the SPSS program (version 18.0.0) (IBM, Corp., Armonk, NY, United States). For multiple comparisons, Fisher’s least significant difference test (LSD) was performed on all data after ANOVA when significant differences (*P* < 0.05) were detected among different treatments. Each experiment was completed with at least three replicates.

## Results

### Effects of Eugenol and Anise Oil on the Thermotolerance of Tomato Plants

**FIGURE 1:**
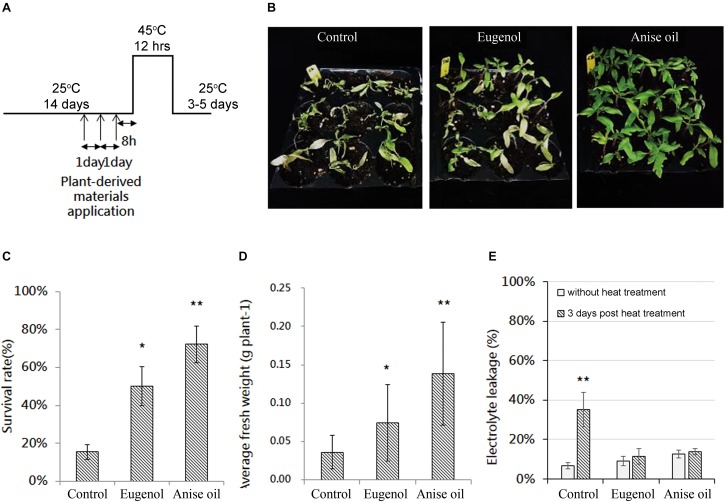
Effects of the pre-application of eugenol and anise oil on the thermotolerance of tomato plants. **(A)** Schedule for the application of plant-derived materials, heat treatment, and harvest. Phenotypes were analyzed at the indicated time points. The concentrations of the plant-derived materials, eugenol and anise oil, were 200 μg mL^–1^. After an exposure to heat stress and a 5-day recovery period, tomato plants with different treatments were photographed **(B)**. The survival rates **(C)** and fresh weights **(D)** of these plants were also recorded based on 18 technical replicates in three independent experiments. Additionally, the relative leakage of leaf electrolytes **(E)** was measured after a 3-day recovery period. Bars represent the mean of three experiments ( ± standard error of the mean). Significant differences between control and anise oil- or eugenol-treated plants were assessed with Student’s *t*-test (^*^P < 0.05; ^∗∗^*P* < 0.01).

Several natural chemicals and synthetic agents have been reported to directly or indirectly influence plants that are exposed to environmental stresses. To assess the effects of eugenol and anise oil on tomato seedlings under heat stress conditions, 12-day-old tomato seedlings were sprayed with control solution, eugenol (200 μg mL^–1^), or anise oil (200 μg mL^–1^) three times daily. At 8 h after the final spray treatment, plants were subjected to heat stress at 45°C for 12 h (Figure 1A). After a 3- or 5-day recovery period at 25°C, the phenotypes, survival rates, and average fresh weights were recorded (Figures 1B–D). Plants treated with eugenol or anise oil grew well after the heat treatment and 5-day recovery period, particularly those treated with anise oil (Figure 1B). In contrast, control plants exhibited significant wilting and yellowing of leaves (Figure 1B). Additionally, the survival rates (Figure 1C) and average fresh weights (Figure 1D) were significantly greater for the eugenol- and anise oil-treated plants than the control. Without heat treatment, the plants treated with eugenol, anise oil and control solution showed similar healthy phenotypes (Supplementary Figure S1B).

A previous study has reported that heat alters membrane fluidity and lipid peroxidation and also impairs membrane selectivity (Dias et al., 2010). To assess whether the priming agents affected membrane stability, electrolyte leakage was analyzed for untreated (25°C) and heat-treated plants after a 3-day recovery period. Electrolyte leakage was significantly lower in eugenol- and anise oil-treated plants than control (Figure 1E). These results suggest that eugenol and anise oil treatments inhibit electrolyte leakage in tomato plants.

**FIGURE 2:**
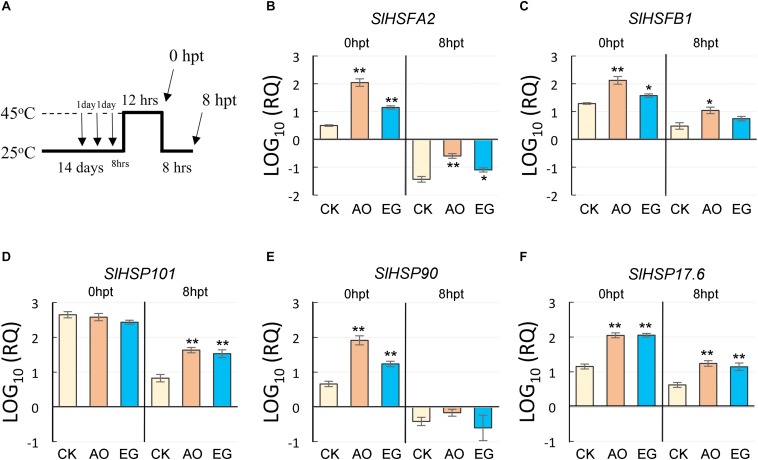
Effects of anise oil and eugenol on the expression of HSFs and HSPs in tomato plants under heat stress. Fourteen-day-old tomato seedlings treated with a control solution (CK), anise oil (AO), or eugenol (EG) for 24 h were subjected to heat stress at 45°C for 12 h. After heat treatment (0 hpt) and 8-h recovery (8 hpt), total RNA was extracted from these plants **(A)**. The *SlHSFA2* **(B)**, *SlHSFB1* **(C)**, *SlHSP101* **(D)**, *SlHSP90* **(E)**, and *SlHSP17.6* **(F)** expression levels were analyzed by RT-qPCR. The *β-actin* expression level was used as the internal control. Relative gene expression levels were normalized against the expression level in untreated tomato plants. Data were analyzed with the 2^–Δ⁢Δ⁢*Ct*^ method. Bars represent the mean ( ± standard error of the mean) of three independent biological replicates each with three technical replicates. Significant differences between control and anise oil- or eugenol-treated plants were assessed with Student’s *t*-test (^*^P < 0.05; ^∗∗^*P* < 0.01).

Heat shock proteins and HSFs are considered the central components of heat stress response in plants (Nover and Scharf, 1997; Kotak et al., 2007). Thus, *SlHSFA2*, *SlHSFB1*, *SlHSP101*, *SlHSP90*, and *SlHSP17.6* were analyzed by RT-qPCR (Figure 2); 12-day-old tomato seedlings sprayed with control solution, eugenol (200 μg mL^–1^), or anise oil (200 μg mL^–1^) three times daily were incubated at 45°C for 12 h (Figure 2A). After heat treatment (0 hpt) and 8-h recovery (8 hpt), total RNA from those seedlings was isolated and analyzed. At 0 hpt, *SlHSFA2, SlHSFB1, SlHSP90*, and *SlHSP17.6* were significantly increased in anise oil- and eugenol-treated plants compared to the control (Figures 2B,C,E,F). At 8 hpt, the expression levels of *SlHSP101* and *SlHSP17*.6 were significantly higher in anise oil- and eugenol-treated plants than in the control (Figures 2D,F). This suggests that eugenol and anise oil regulate the expression of *HSF* and *HSP* genes under heat stress. In addition, the expression of genes in the RNA silencing pathway and antioxidant was also analyzed (Supplementary Figure S2). The expression levels of *SlDCL2* and *SlAPX2* were significantly higher in anise oil- and eugenol-treated plants than in the control at 8 hpt (Supplementary Figures S2B,D), while the expression of *SlAGO2A*, *SlDCL4*, and *SlCAT2* did not significantly differ among plants irrespective of the treatments (Supplementary Figures S2A,C,E). Taken together, these results suggest that priming tomato plants with eugenol or anise oil enhances thermotolerance in plants.

### Exogenous Application of Anise Oil and Eugenol Increased the Resistance of Tomato Plants Against TYLCTHV

**FIGURE 3:**
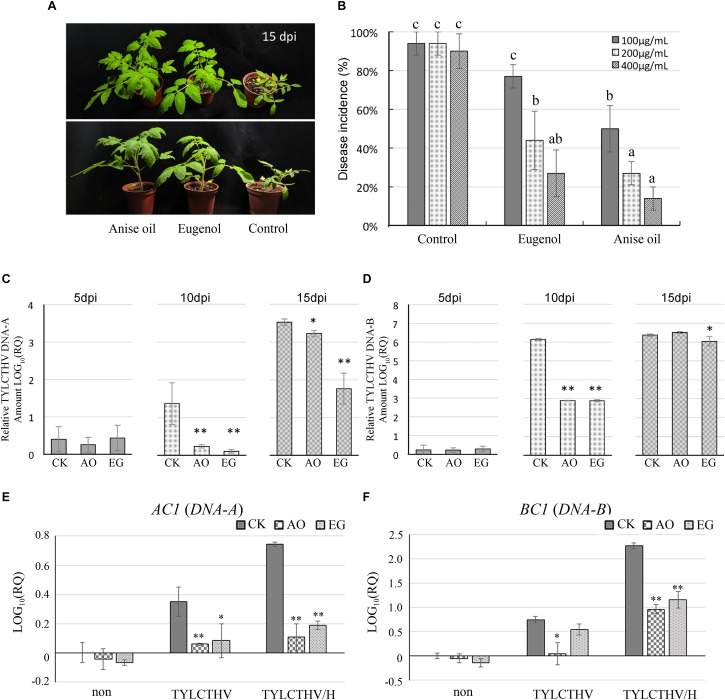
Antiviral effects of anise oil and eugenol on TYLCTHV. Tomato plants were treated with foliar applications of eugenol (100, 200, or 400 μg mL^–1^) or anise oil (100, 200, or 400 μg mL^–1^) 24 h before TYLCTHV inoculations. The plants were photographed at 15 days post-inoculation (dpi) **(A)**. The disease incidence was evaluated by detecting TYLCTHV-positive plants at 15 dpi. Fisher’s least significant difference test (LSD) was employed examining significant differences (*P* < 0.05) among different treatments. **(B)**. Additionally, the relative abundances of TYLCTHV DNA-A **(C)** and DNA-B **(D)** in anise oil- and eugenol-treated plants and control were estimated by a quantitative real-time PCR assay. Samples were collected every 5 days after the onset of the viral infection (0, 5, 10, and 15 dpi). Five individual samples for each treatment were analyzed. Relative TYLCTHV DNA amounts in tomato plants were normalized against the DNA content at 0 dpi. In addition, relative expression of viral RNA, *AC1* **(E)** and *BC1* **(F)**, at 5 dpi was also analyzed by RT-qPCR. The *β-actin* expression level was used as the internal control. Relative gene expression levels were normalized against the expression level in control. Data were analyzed with the 2^–Δ⁢Δ⁢*Ct*^ method. Bars represent the mean ( ± standard error of the mean) of at least three independent biological replicates each with three technical replicates. Significant differences between control and anise oil- or eugenol-treated plants were assessed with Student’s *t*-test (^*^P < 0.05; ^∗∗^*P* < 0.01).

Several studies have indicated that heat stress and TYLCV infection can induce similar responses in plants (Clarke et al., 2004, 2009; Anfoka et al., 2016; Li et al., 2017). Since TYLCTHV is one of the predominant begomoviruses infecting tomato in Taiwan, tomato plants were infected with TYLCTHV to verify the similarity between plant responses to heat stress and viral disease. Tomato plants were treated with a foliar application of anise oil or eugenol 24 h before exposure to viruliferous whiteflies. At 15 days post-inoculation (dpi), only mild symptoms were observed on seedlings treated with anise oil or eugenol (Figure 3A), whereas severe TYLCTHV-induced disease symptoms, such as leaf yellowing, leaf curling, and stunted growth, were observed in control plants (Figure 3A). Disease incidence at 15 dpi was evaluated based on PCR amplification of TYLCTHV sequences. Exogenous application of eugenol (100, 200, and 400 μg mL^–1^) significantly decreased disease incidence compared to the control (Figure 3B). Similarly, 100, 200, and 400 μg mL^–1^ anise oil treatments also decreased disease incidence in a concentration-dependent manner (Figure 3B). To calculate the amount of TYLCTHV in infected tissues, a qPCR assay was used, targeting the DNA-A and DNA-B genomic segments (Figures 3C,D). DNA-A and DNA-B levels were substantially higher in the control than in anise oil- and eugenol-treated plants at 10 dpi (Figures 3C,D). Furthermore, although the relative DNA-A abundance was elevated among all treatments, virus accumulation in the control was still higher than in anise oil- and eugenol-treated plants at 15 dpi (Figure 3C).

To verify that anise oil and eugenol affected the accumulation of TYLCTHV in tomato plants, the expression levels of *AC1* (replicase-associated protein) and *BC1* (movement protein) were measured by RT-qPCR. *AC1* and *BC1* are located in DNA-A and DNA-B of TYLCTHV, respectively. After TYLCTHV infection, anise oil and eugenol decreased the expression of *AC1* and *BC1* compared to the control (Figures 3E,F). Moreover, heat stress has been demonstrated to enhance accumulation of TYLCV (Anfoka et al., 2016). Anise oil and eugenol also decreased the expression of *AC1* and *BC1* compared to the control after TYLCTHV infection combined with heat treatment (Figures 3E,F).

### Exogenous Application of Anise Oil and Eugenol Upregulated the Expression of Salicylic Acid- and Jasmonic Acid- Mediated Genes

**FIGURE 4:**
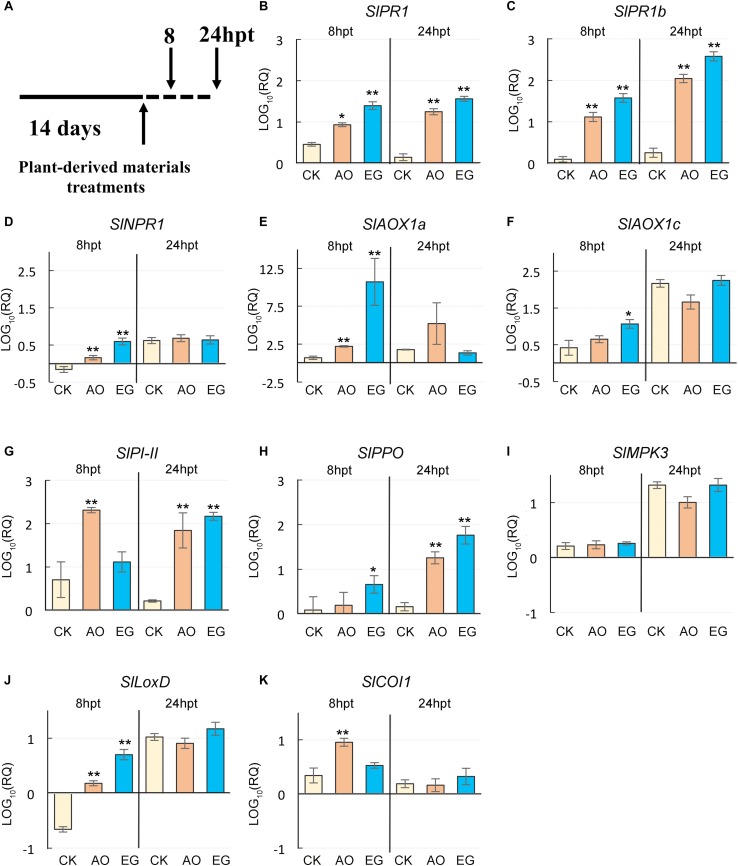
Effects of anise oil and eugenol on the expression of SA- and JA-related genes in tomato plants. Fourteen-day-old tomato seedlings were treated with a control solution (CK), anise oil (AO), or eugenol (EG). Total RNA was extracted from these plants at 8 h post-treatment (hpt) and 24 hpt **(A)**. The expression levels of SA-related defense genes, *SlPR1* **(B)**, *SlPR1b* **(C)**, *SlNPR1* **(D)**, *SlAOX1a* **(E)**, and *SlAOX1c* **(F)**, and JA-related defense genes, *SlPI-II* **(G)**, *SlPPO* **(H)**, *SlMAPK3* **(I)**, *SlLoxD* **(J)**, and *SlCOI1* **(K)**, were analyzed by qRT-PCR. The *β-actin* expression level was used as the internal control. Relative gene expression levels were normalized against the expression level in untreated tomato plants. Data were analyzed with the 2^–Δ⁢Δ⁢*Ct*^ method. Bars represent the mean ( ± standard error of the mean) of three independent biological replicates each with three technical replicates. Significant differences between control and anise oil- or eugenol-treated plants were assessed with Student’s *t*-test (^*^P < 0.05; ^∗∗^*P* < 0.01).

Since SA and JA enhance plant tolerance to heat and viral infections (Clarke et al., 2004; Shang et al., 2011; Li et al., 2017; Makarova et al., 2018), we measured the expression level of selected SA- and JA-related genes in anise oil- and eugenol-treated plants (Figure 4). The RT-qPCR results revealed that the expression levels of the SA-associated genes, *SlPR1*, *SlPR1b*, *SlNPR1*, *SlAOX1a*, and *SlAOX1c*, were significantly upregulated in eugenol and anise oil treatment compared to the control at 8 or 24 hpt (Figures 4B–F). Additionally, the expression levels of the JA-associated genes, *SlPI-II*, *SlPPO*, *SlLoxD*, and *SlCOI1*, were also strongly upregulated in the plants treated with anise oil or eugenol compared to the control at 8 or 24 hpt (Figures 4G–K). The expression of *SlMPK3* did not significantly differ among plants irrespective of the treatments (Figure 4I). Overall, eugenol and anise oil significantly increased the expression of a few SA- and JA-related genes to modulate stress responses.

### Exogenous Application of Anise Oil and Eugenol Upregulated the Expression of Genes for RNA Silencing Machinery

**FIGURE 5:**
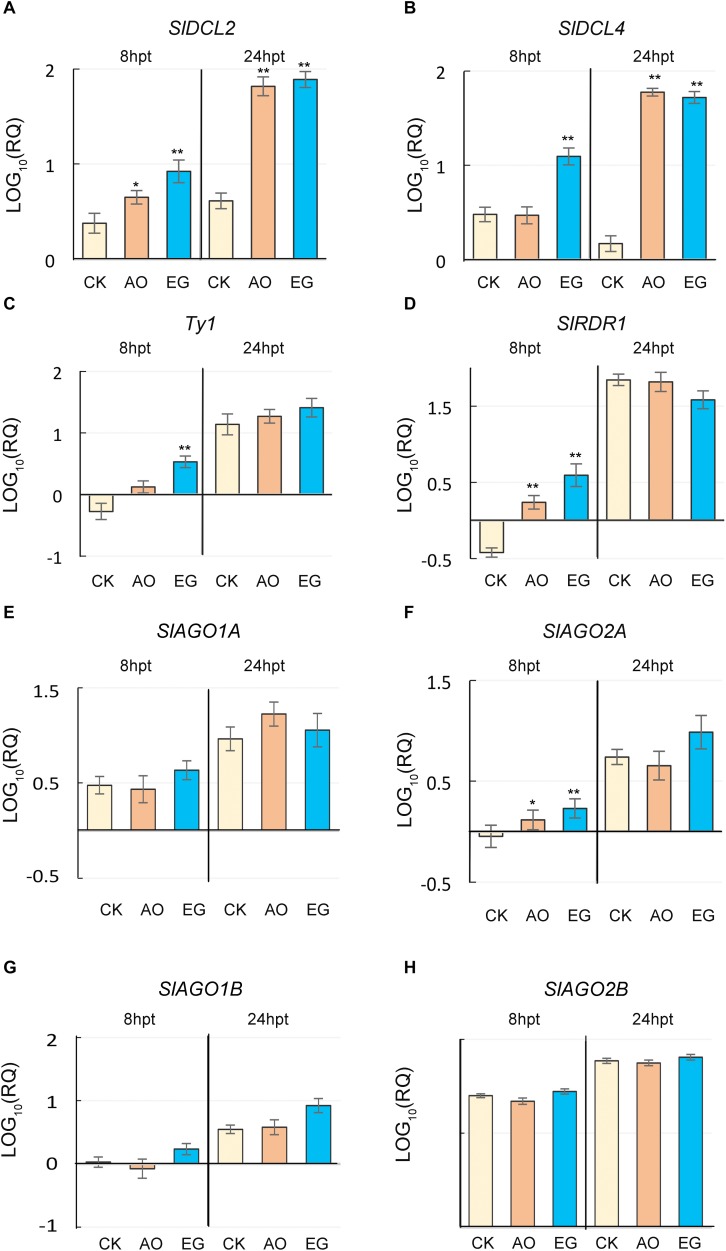
Effects of anise oil and eugenol on the expression of RNA silencing genes in tomato plants. Fourteen-day-old tomato seedlings were treated with a control solution (CK), anise oil (AO), or eugenol (EG). Total RNA was extracted from these plants at 8 h post-treatment (hpt) and 24 hpt. The expression levels of RNA silencing genes, *SlDCL2* **(A)**, *SlDCL4* **(B)**, *Ty-1* **(C)**, *SlRDR1* **(D)**, *SlAGO1A* **(E)**, *SlAGO2A* **(F)**, *SlAGO1B* **(G)**, and *SlAGO2B* **(H)** were analyzed by RT-qPCR. The *β-actin* expression level was used as the internal control. Relative gene expression levels were normalized against the expression level in untreated tomato plants. Data were analyzed with the 2^–Δ⁢Δ⁢*Ct*^ method. Bars represent the mean ( ± standard error of the mean) of three independent biological replicates each with three technical replicates. Significant differences between control and anise oil- or eugenol-treated plants were assessed with Student’s *t*-test (^*^P < 0.05; ^∗∗^*P* < 0.01).

To elucidate if exogenous application of anise oil and eugenol influences genes involved in RNA silencing machinery, genes reported to be involved in the RNA silencing response against TYLCV were analyzed. *SlDCL2*/*SlDCL4*-silenced tomato plants were shown to be less resistant against TYLCV (Li et al., 2018). *Ty-1*/*Ty-3*, a DFDGD-Class RNA-dependent RNA polymerase (RDR), mediates resistance against TYLCV (Verlaan et al., 2013; Butterbach et al., 2014). Hence, the analysis was focused on the gene expression levels of *SlDCL2*, *SlDCL4*, and *Ty-1* (Figures 5A–C). At 24 hpt, *SlDCL2* and *SlDCL4* transcript levels were higher in anise oil and eugenol treatments compared to the control (Figures 5A,B). At 8 hpt, *SlDCL2* transcript levels were also higher in anise oil and eugenol treatments compared to the control, whereas *SlDCL4* transcript levels were higher only in eugenol-treated plants (Figures 5A,B). In case of *Ty-1*, transcript levels showed a slight increase after anise oil and eugenol treatments compared to the control at 8 hpt; conversely, the transcripts showed no significant differences among all treatments at 24 hpt (Figure 5C). The expression of other genes in the RNA silencing pathway was also analyzed (Figures 5D–H). *SlAGO1A*, *SlAGO1B*, and *SlAGO2B*, transcript levels were not altered at 8 or 24 hpt in all treatments (Figures 5E,G,H). Only *SlAGO2A* and *SlRDR1* transcripts showed significant increases at 8 hpt (Figures 5D,F). Therefore, these results reveal that eugenol and anise oil may act as priming agents to up-regulate specific RNA silencing pathway genes.

### Exogenous Application of Anise Oil and Eugenol Altered the Transcriptional Activity of TYLCTHV and Expression of Defense Genes Under Stress

**FIGURE 6:**
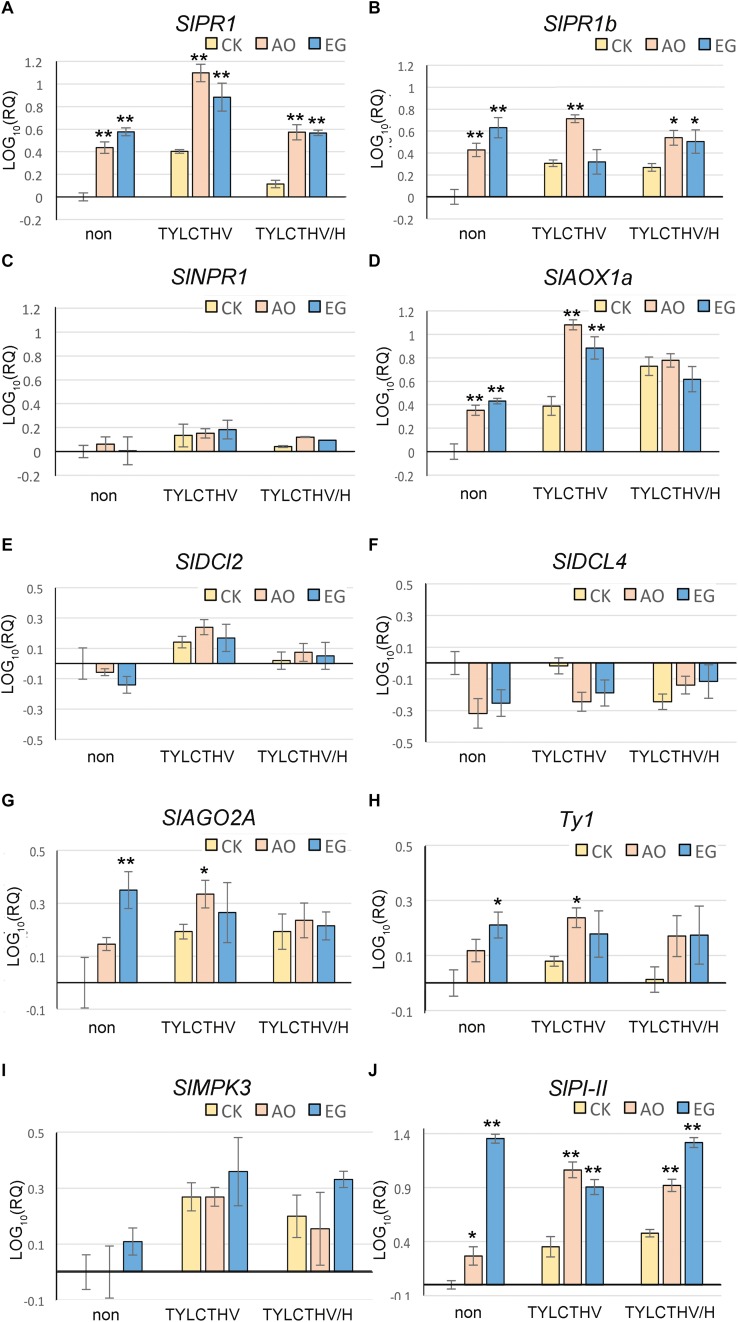
Effects of anise oil and eugenol on the expression of defense-related genes in tomato plants under TYLCV infection with/without heat stress. Fourteen-day-old tomato seedlings treated with a control solution (CK), eugenol (EG), or anise oil (AO) for 24 h were inoculated TYLCTHV. Then, some plants were incubated at 45°C for 12 h. Total RNA was extracted from these plants. The expression levels of SA-related defense genes, *SlPR1* **(A)**, *SlPR1b* **(B)**, *SlNPR1* **(C)**, and *SlAOX1a* **(D)**, RNA silencing genes, *SlDCL2* **(E)**, *SlDCL4* **(F)**, *SlAGO2A* **(G)**, and *Ty-1* **(H)**, and JA-related defense genes, *SlPI-II* **(I)** and *SlMAPK3* **(J)**, were analyzed by RT-qPCR. The *β-actin* expression level was used as the internal control. Relative gene expression levels were normalized against the expression level in control. Data were analyzed with the 2^–Δ⁢Δ⁢*Ct*^ method. Bars represent the mean ( ± standard error of the mean) of three independent biological replicates each with three technical replicates. Significant differences between control and anise oil- or eugenol-treated plants were assessed with Student’s *t*-test (^*^P < 0.05; ^∗∗^*P* < 0.01).

Anise oil and eugenol can affect the expression levels of some of SA-, JA-, and RNA silencing pathway genes (Figures 4, 5). Therefore, some defense-related genes were also analyzed for the effect of TYLCTHV infection with/without heat stress (Figure 6). The results showed that the expression levels of *SlPR1*and *SlPR1b* were significantly higher after anise oil and eugenol treatments under TYLCTHV/heat stress (Figures 6A,B). *SlAOX1a* expression was upregulated by anise oil and eugenol under TYLCTHV infection compared to the control (Figure 6D). In addition, *SlPI-II* was also increased by TYLCTHV infection and TYLCTHV/heat stress (Figure 6J). *Ty1* was upregulated under TYLCTHV/heat stress by eugenol treatment and was upregulated under TYLCTHV infection by anise oil treatment (Figure 6H). With TYLCTHV infection and TYLCTHV/heat stress, the expression levels of *SlNPR1*, *SlDCL2*, *SlDCL4*, and *SlMPK3* showed no significant differences among anise oil, eugenol and control (Figures 6C,E,F,I). These results suggest that genes, such as *SlPR1*, *SlPR1b*, and *SlPI-II*, were upregulated in anise oil- and eugenol-treated plants during both priming (Figures 4B,C,G) and stresses-induced states (Figures 6A,B,J). Moreover, plants pre-treated with anise oil and eugenol resisted TYLCTHV infection under normal temperature and heat stress. Therefore, these results suggest that anise oil- and eugenol-treated plants may regulate a number of defense genes to mediate TYLCTHV resistance.

### Exogenous Application of Anise Oil and Eugenol Modulated the Endogenous Salicylic Acid and Jasmonic Acid Contents in Unstressed Tomato Plants

**FIGURE 7:**
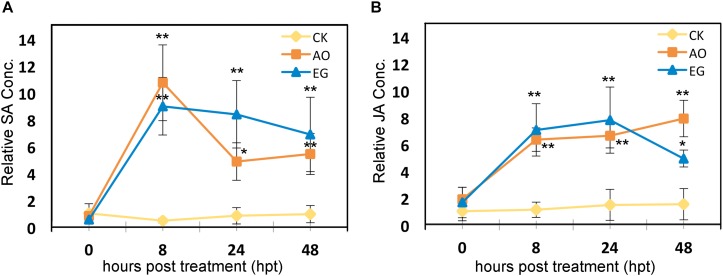
Effects of anise oil and eugenol on the endogenous SA and JA contents. Fourteen-day-old tomato seedlings were treated with a control solution (CK), eugenol (EG), or anise oil (AO). The treated leaves were harvested just before the treatments (0 h) and at 8, 24, and 48 h after treatments. The endogenous SA **(A)** and JA **(B)** contents were detected. The SA and JA levels of the untreated tomato plants was treated as the normalized reference, with a value of one. Bars represent the mean ( ± standard deviation) from two measurements (each with triplicate samples). Significant differences between control and anise oil- or eugenol-treated plants were assessed with Student’s *t*-test (^*^P < 0.05; ^∗∗^*P* < 0.01).

To determine whether endogenous SA and JA contents correspond to the expression patterns of SA- and JA-mediated genes, we examined the endogenous SA and JA contents in tomato plants sprayed with control solution, eugenol, or anise oil. Both SA (Figure 7A) and JA (Figure 7B) levels increased significantly in anise oil- and eugenol-treated plants compared to the control. TYLCV infections are usually accompanied by heat stress in agricultural systems (Czosnek, 2007). Heat also aggravated TYLCV accumulation in plants after TYLCV infection (Anfoka et al., 2016). Hence, the endogenous SA and JA contents were also examined in tomato plants treated with heat and TYLCTHV/heat. Under these stresses, the endogenous SA and JA contents showed no significant difference between control, eugenol, and anise oil treatments (Supplementary Figures S3A,B). Overall, these results suggest that eugenol and anise oil may have some effect to modulate the endogenous SA and JA contents in unstressed plants.

### Exogenous Application of Anise Oil and Eugenol Altered the Amounts of H_2_O_2_ in Tomato Leaves

**FIGURE 8:**
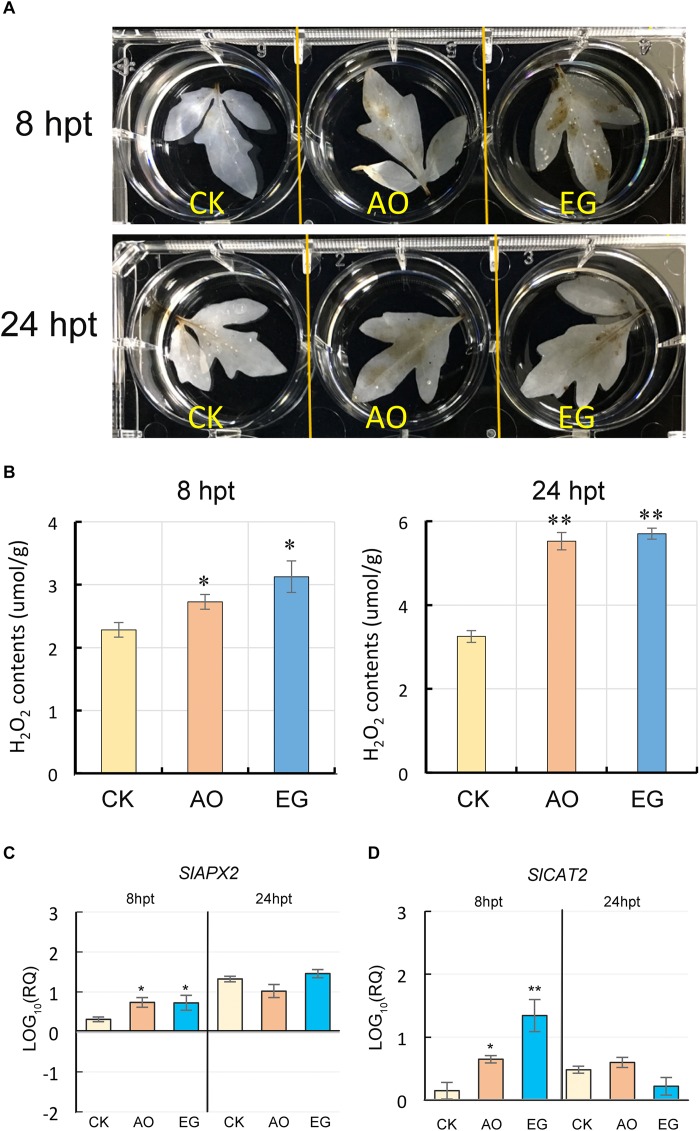
Effects of anise oil and eugenol on the accumulation of H_2_O_2_ and the expression of antioxidant genes in tomato plants. 3′-Diaminobenzidine (DAB) staining was used to detect H_2_O_2_ in control, anise oil-, and eugenol-treated tomato plants at 8 hpt and 24 hpt **(A)**. Furthermore, H_2_O_2_ content was quantified by titanium chloride method **(B)**. In addition, expression of antioxidant genes, *SlAPX2* **(C)** and *SlCAT2* **(D)**, was also analyzed by RT-qPCR. The *β-actin* expression level was used as the internal control. Relative gene expression levels were normalized against the expression level in untreated tomato plants. Data were analyzed with the 2^–Δ⁢Δ⁢*Ct*^ method. Bars represent the mean (± standard error of the mean) of three independent biological replicates each with three technical replicates. Significant differences between control and anise oil- or eugenol-treated plants were assessed with Student’s *t*-test (^*^P < 0.05; ^∗∗^*P* < 0.01).

A rapid ROS burst is involved in early plant defense responses (Kadota et al., 2015). Among the ROS, H_2_O_2_ produced by the chloroplast is an important signaling molecule in response to diverse environmental stresses (Perez-Ruiz et al., 2006). There is evidence that SA induces the accumulation of H_2_O_2_, thereby increasing oxidative stress (Chao et al., 2010), and the increasing H_2_O_2_ level may also lead to SA biosynthesis (Leon et al., 1995). When SA and H_2_O_2_ accumulate to a high level, the local PR expression levels may also increase (Devadas and Raina, 2002). In this study, we examined whether the application of anise oil or eugenol affects the balance of ROS in tomato plants. Both anise oil and eugenol induced rapid generation of H_2_O_2_ in tomato leaves at 8 and 24 hpt as indicated by the dark color in bleached leaves (Figure 8A). Furthermore, the titanium chloride method to quantify H_2_O_2_ revealed that anise oil and eugenol treatments could stimulate the generation of H_2_O_2_ in tomato leaves compared to the control (Figure 8B). In addition, expression of the antioxidant-associated genes, SlAPX2 and SlCAT2, was also altered by anise oil and eugenol (Figures 8C,D). Overall, anise oil and eugenol may increase H_2_O_2_ accumulation to contribute to stress responses.

## Discussion

Some plant-derived materials reportedly enhance plant resistance against stresses (Bishop, 1995; Ertani et al., 2013; Vergnes et al., 2014). Eugenol, a natural plant-derived compound, may function as an anti-TYLCV agent in tomato plants (Wang and Fan, 2014; Sun et al., 2016). In this study, thermotolerance-related phenotypes, including increased average fresh weight and survival rates, were observed in plants treated with exogenous anise oil or eugenol (Figure 1). Eugenol (Figures 2, 4, 7; Wang and Fan, 2014) and anise oil (Figures 2, 4, 7) appear to increase the SA content and upregulate the expression of a few downstream genes. Under heat stress, SA can influence the thermotolerance of crop plants (Larkindale et al., 2005; Wang et al., 2010; Khan et al., 2013). In *Arabidopsis*, SA regulates *HSP17.6* to promote thermotolerance (Clarke et al., 2004). Anise oil and eugenol regulate the expression of *HSF*s and *HSP*s under heat stress (Figure 2). Thus, these findings suggest that eugenol and anise oil may act as priming agents and elevate the SA content to mediate the thermotolerance of tomato plants.

Tomato yellow leaf curl Thailand virus is the predominant tomato-infecting begomovirus in Taiwan (Tsai et al., 2011). Due to the lack of efficient priming agents, it is difficult to control TYLCTHV in the field. Previous studies have shown that eugenol is more effective as a protective agent than as a curative agent against TYLCV (Wang and Fan, 2014; Sun et al., 2016). Our results suggest that eugenol confers resistance not only to TYLCV but also TYLCTHV (Figure 3). We also observed that anise oil increases the resistance of tomato plants to TYLCTHV infections (Figure 3). The TYLCTHV incidence decreased by 75 and 60% following anise oil and eugenol (200 μg mL^–1^) treatments, respectively (Figure 3B). Our TYLCTHV DNA quantification (Figures 3C,D) showed that pre-treatment with anise oil or eugenol significantly reduced the accumulation rate of both TYLCTHV DNA-A and DNA-B. Consistent with the data provided in published reports, our qPCR analysis revealed that anise oil and eugenol inhibit the accumulation of DNA-A to restrict TYLCTHV infection in seedlings (Figure 3C). Even though relative DNA-B amount showed no difference between these three treatments at 15 dpi, the lower DNA-A accumulation rate may have mitigated the symptom development (Figures 3A,C,D). In addition, the amounts of TYLCTHV DNA-B reached saturation more quickly in the control than in anise oil- and eugenol-treated plants (Figure 3D). DNA-B of some bipartite begomoviruses, such as ToLCNDV also reached saturation at 10–14 dpi and showed a comparatively higher accumulation level than DNA-A (Jyothsna et al., 2013). This implies that TYLCTHV DNA-B has a higher accumulation rate than DNA-A regardless of treated plants or control plants (Figures 3C,D). Taken together, these findings suggest that the delayed accumulation of DNA-A and DNA-B in seedlings treated with anise oil or eugenol is responsible for mitigating the TYLCTHV disease and restricting the development of the characteristic symptoms.

Salicylic acid contributes to plant resistance against viral diseases and heat stress (Dat et al., 1998, 2000; Larkindale and Knight, 2002). Accordingly, the genes associated with thermotolerance and SAR defense responses have overlapping expression patterns (Clarke et al., 2004). The transcription of SA-related *PR* defense genes is heat inducible and can be triggered by eugenol in response to TYLCV (Wang and Fan, 2014). A primed state induced by SA includes the activation of the mitogen activated kinase cascade, up-regulation of *NPR1*, and increased transcripts of *PR* defense genes (Kohler et al., 2002; Yi et al., 2015). Here, elevated levels of *SlNPR1*, *SlPR1, SlPR1b*, and *SlAOX1a* transcripts were recorded in anise oil- and eugenol-treated plants at 8 or 24 hpt (Figures 4B–E). This suggests that anise oil and eugenol may be involved in the induction of SA responses. In addition, several studies have indicated that SA-mediated defense and RNA silencing may be connected (Baebler et al., 2014; Campos et al., 2014; Lee et al., 2016). SA can induce *AGO2*, *AGO3*, *RDR1*, *DCL2* and *DCL4*, but has no effect on most of *AGO*s or *RDRs* in *Arabidopsis* seedlings (Alazem et al., 2019). After the application of exogenous SA and inoculation with TYLCV, *SlDCL2* and *SlDCL4* expressions were significantly upregulated (Li et al., 2018). Tomato plants silenced for *SlDCL2*-, *SlDCL4*-, and *SlDCL2/4*- showed increased susceptibility to TYLCV and reduced levels of *SlPR1* and *SlPR1b* (Li et al., 2018). In this study, *SlDCL2* and *SlDCL4* transcript levels were increased in anise oil- and eugenol-treated plants compared to the control (Figures 5A,B). *DCL*s, *AGO*s, and *RDR*s are crucial components in the RNA silencing machinery. However, the slightly increased *Ty-1*, *SlRDR1*, and *SlAGO2A* transcripts were recorded only at 8-h after anise oil and eugenol treatments (Figures 5C,D,F). These results raise the possibility that there are multiple factors affecting the RNA silencing pathway when plants are primed with anise oil and eugenol.

Reactive oxygen species have been implicated in mediating plant systemic signals in response to biotic and abiotic stresses (Gilroy et al., 2016). In the defense against viruses, SA induces an oxidative burst via the accumulation of H_2_O_2_, which may induce SAR (Chen et al., 1993; Nie et al., 2015). Respiratory burst oxidase homolog-dependent ROS can be activated by heat stress and may subsequently function as long-distance signals (Suzuki and Katano, 2018). Increased SA and H_2_O_2_ contents in anise oil- and eugenol-treated plants were observed in our study (Figures 7A, 8A,B). Plant resistance to TMV was demonstrated by the SA-dependent pathway with the *AOX* gene (Kachroo et al., 2000). Accordingly, *SlAOX1a*, and *SlAOX1c* expression were significantly upregulated at 24-h after anise oil and eugenol treatments (Figures 4E,F). This up-regulation was positively correlated with the accumulation of SA and H_2_O_2_ (Figures 7A, 8A,B). Thus, anise oil and eugenol enhanced the resistance of tomato plants to TYLCTHV and heat stress by activating SA-dependent signaling pathways.

Jasmonate is also an important viral defense hormone in plants (Alazem and Lin, 2015, 2017) and functions cooperatively with SA to confer basal thermotolerance in *A. thaliana* (Sharma and Laxmi, 2016). Interestingly, JA-mediated defenses against TYLCV seem to be more powerful than SA-mediated defenses in terms of viral transmission efficiency (Zarate et al., 2007; Li R. et al., 2014). The JA signaling pathway regulates the production of several plant secondary metabolites and defensive proteins that are harmful to herbivores (Zarate et al., 2007; Yang et al., 2008; Zhang et al., 2012). The suppression of JA-mediated defenses against *B. tabaci*, which is the vector for TYLCV transmission, may increase the number of viruliferous vectors produced and promote the transmission of the virus to new hosts (Su et al., 2016). TYLCCNV can promote the performance of whiteflies in infected tobacco plants by interfering with MYC2 dimerization and inhibiting the JA signaling pathway (Li R. et al., 2014). Additionally, TYLCV enhances the performance of whiteflies in infected tomato plants by downregulating the expression of the JA-regulated defense gene *SlPI-II* (Sun et al., 2017). In this study, the expression of *SlPPO-F* and *SlPI-II* was upregulated by anise oil and eugenol treatments (Figures 4G,H). Similarly, JA contents also increased following anise oil and eugenol treatments (Figure 4B), which is consistent with the expression-level changes of the downstream genes. Therefore, these results suggest that the pre-application of anise oil or eugenol may alleviate TYLCTHV infection in tomato plants via whiteflies by modifying the JA content and the expression of JA-related genes.

Anise oil- and eugenol-activated SA- and JA-dependent signaling pathways were confirmed at the priming state due to the similar levels of SA and JA among both treatments under heat stress and TYLCTHV infection (Supplementary Figures S3A,B). Virus infection and temperature stress can interfere with the homeostatic balance, thus causing the accumulation of ROS in the apoplast (Awasthi et al., 2015; Carmo et al., 2017). This increase in ROS possibly activates plant defense response and overrides some of the priming effects caused by plant-derived materials. Phytohormones appear to form a central hub that links, integrates, and re-programs multiple stress responses (Golldack et al., 2014). In this study, anise oil and eugenol enhanced the resistance of tomato plants to TYLCTHV infection and heat stress conditions at the priming state by activating two crucial defense hormones SA and JA. These two hormones and some of their downstream genes play a major role in plant defenses against TYLCV and heat stress (Clarke et al., 2004, 2009; Wang and Fan, 2014; Sun et al., 2016; Li et al., 2017).

The regulatory network underlying plant responses to stresses is much more complex than previously assumed. Even though previous studies showed that SA and JA are antagonistic hormones, there is increasing evidence that SA and JA do not have antagonistic activity against each other under some circumstances, including heat stress and TYLCV infection (Clarke et al., 2009; Su et al., 2016; Wang et al., 2017). TYLCV was found to suppress JA-mediated responses to *B. tabaci* via SA-independent mechanisms (Su et al., 2016). In this study, we have shown that anise oil and eugenol can trigger SA- and JA-defense mechanisms to reduce the accumulation of TYLCTHV and enhance the heat stress tolerance of tomato plants.

## Author Contributions

W-AT and J-SL developed the research concept, and wrote the manuscript. W-AT and S-HW performed most of the experiments. M-CC contributed to some experiments and revised the manuscript. W-ST provided the experimental materials and revised the manuscript. J-SL supervised the entire study. All authors have read and approved the final manuscript.

## Conflict of Interest Statement

The authors declare that the research was conducted in the absence of any commercial or financial relationships that could be construed as a potential conflict of interest.

## Significance Statement

Plant-derived materials, eugenol and anise oil, may enhance tomato thermotolerance and restrict tomato yellow leaf curl Thailand virus replication by regulating salicylic acid- and jasmonic acid-mediated defenses.

## Abbreviations

AAP: acquisition access period
AGO: argonaute
APX: ascorbate peroxidase
BTH: benzothiadiazole
DAB, 3: 3-diaminobenzidine
DCLs: dicer-like endoribonucleases
dpi: days post-inoculation
hpt: hours post-treatment
GAPDH: glyceraldehyde-3-phosphate dehydrogenase
HR: hypersensitive response
HSFs: heat stress transcription factors
HSPs: heat shock proteins
IAP: inoculation access period
JA: jasmonic acid
MAPK: mitogen-activated protein kinase
MeJA: methyl jasmonate
PCR: polymerase chain reaction
PPO-F: polyphenol oxidase-F
PR: pathogenesis-related gene
PI-II: proteinase inhibitor II
qPCR: quantitative PCR
RDR: RNA-directed RNA polymerase
ROS: reactive oxygen species
RT-PCR: reverse transcription PCR
SA: salicylic acid
SAR: systemic acquired resistance
TMV: tobacco mosaic virus
ToLCTWV: tomato leaf curl Taiwan virus
TSWV: tomato spotted wilt virus
TYLCCNV: tomato yellow leaf curl China virus
TYLCTHV: tomato yellow leaf curl Thailand virus
TYLCV: tomato yellow leaf curl virus
vsiRNAs: viral small interfering RNAs.
