# Supplementary material for: Priming of Plant Resistance to Heat Stress and Tomato Yellow Leaf Curl Thailand Virus With Plant-Derived Materials
Source: Front Plant Sci. 2019 Jul 12;10:906. doi: 10.3389/fpls.2019.00906 (PMC6640737; doi:10.3389/fpls.2019.00906)
Supplement: Supplementary file 1 [file Table_1.docx]

**Table S1. Primer sets used for amplification of TYLCTHV in this study.**

| Primer name | | Sequence (5’-3’) | Accession No. |
| --- | --- | --- | --- |
| TYLCTHV-specific primer | THAV3 | CCACATCGTCTTYGTTCTG |  |
|  | THAC | CTTAAYYTTRATATTYTCATCCATCCA |  |
| TYLCTHV DNA-A | Forward | gCTATgCAATCggTgTCTgg | GU723742.1 |
|  | Reverse | TggCCgCTTTTggATTTTgA |  |
| TYLCTHV DNA-B | Forward | ACAACCTCTgACgCTgAAgg | GU723754.1 |
|  | Reverse | AAgggCATACTTgggCTTCC |  |
| GAPDH | Forward | gCCACTCAgAAgACCgTTgA | NM_001247874.2 |
|  | Reverse | AggTCAACCACggACACATC |  |

**Table S2. Primer sets used for gene expression analysis in this study.**

| Target gene | | Sequence (5’-3’) | Accession No./ SGN locus |
| --- | --- | --- | --- |
| *AC1* | Forward | GCCCACATCGTCTTCCCCG | GU723742.1 |
|  | Reverse | CTTGTAATAATGTTCTCAGTGCCGCTG |  |
| *BC1* | Forward | ACAACCTCTgACgCTgAAgg | GU723754.1 |
|  | Reverse | AAgggCATACTTgggCTTCC |  |
| *SlPR1b* | Forward | ggTCgggCACgTTgCA | NM_001247385 |
|  | Reverse | gATCCAgTTgCCTACAggACATA |  |
| *SlPR1* | Forward | AACgCTCACAATgCAgCTCgT | NM_001247429.1 |
|  | Reverse | AAggTCCACCAgAgTgTTgC |  |
| *SlNPR1* | Forward | TgTgggAAAgATAgCAgCACg | Solyc07g040690.2 |
|  | Reverse | gTCCACACAAACACACACATC |  |
| *SlAOX1a* | Forward | TCATTACCAAGGACAACAGC | NM_001247188 |
|  | Reverse | GGAACAAAATAGTGACGGAC |  |
| *SlAOX1c* | Forward | TAAGGCGATTCGAGCACA | XM_004244382 |
|  | Reverse | CCATTAAGACAAGTCCACGT |  |
| *SlDCL2* | Forward | gAgTgCCATAATgCACgAgg | Solyc 06g048960 |
|  | Reverse | TTgCCAATAACTATCTgCTgTg |  |
| *SlDCL4* | Forward | CTTTgTTgAACTACCTCCTg | Solyc 07g005030 |
|  | Reverse | ATTgCTAACTCCCTCCC |  |
| *SlRDR1* | Forward | TGCATCCTGGTGATATTCGT | NM_001247390.1 |
|  | Reverse | TCCAAATCACTCCCAGAACA |  |
| *SlTy1* | Forward | GGCAAAATATGCAGCCAGGCTTTCC | XM_010323869 |
|  | Reverse | TCAGTATGTATACGAGGTTCGCCGT |  |
| *SlAGO1A* | Forward | GTGGAATAGCCCCTCAACAGTC | NM_001279128 |
|  | Reverse | TTGGTTCAGGTGGCTGAGATG |  |
| *SlAGO1B* | Forward | TATCAGCAGGGCAGAGGGACT | NM_001279332 |
|  | Reverse | CTTCCACAAGTAATGGGCTGAG |  |
| *SlAGO2A* | Forward | GGACGGATGTTTCAAGAGGT | NM_001279306 |
|  | Reverse | GATCAACAAGGTGGCATCAG |  |
| *SlAGO2B* | Forward | GCGATGCACTAAACCTGTCT | XM_010318699 |
|  | Reverse | CTGGGAATACATAATACGGGA |  |
| *SlPI-II* | Forward | TgATgAACCCAAggCAAATA | K03291 |
|  | Reverse | ACACAACTTgATgCCCACAT |  |
| *SlPPO-F* | Forward | CggAgTTTgCAgggAgTTATAC | AK_247126.1 |
|  | Reverse | TTgATCTCCACACTTTCAATgg |  |
| *SlLOXD* | Forward | CCATCCTCACCACCCTCATC | Solyc03g122340.2 |
|  | Reverse | TACTCgggATCgTTCTCgTC |  |
| *SlCOI1* | Forward | TTCTgCATATTTCTCgTTCCTgCAA | Solyc05g052620.2 |
|  | Reverse | gCTAgTTCATgTgCCCATTCTCCAT |  |
| *SlMAPK3* | Forward | CATgATgCAgCTgATgAACCA | Solyc06g005170.2 |
|  | Reverse | gCATATTCAggATTCAACgCC |  |
| *SlCAT2* | Forward | ATTTGGTGGAGAAACTTGCC | AF112368 |
|  | Reverse | CTGTACACCAGGAGCTCGAA |  |
| *SlAPX2* | Forward | TgggAgggTggTgACATATTTT | DQ099421 |
|  | Reverse | TTgAAgTgCATAACTTCCCATCTTT3 |  |
| *SlHsfA2* | Forward | ACCTTGTGGATCAGCTTGGTTTCC | CAA47870 |
|  | Reverse | AATAGTGGAGGAGGCCAGAGGAAC |  |
| *SlHSP17C* | Forward | ACTTggCATCgTgTggAACg | AJ225046 |
|  | Reverse | TgATCCATCTTTgCgTTCTCTgg |  |
| *SlHsfB1* | Forward | GGTGCAGG CGAAGAAACAATGC | CAA39034 |
|  | Reverse | TCATATCGGGTGCAACCTTCACG |  |
| *SlHsp101* | Forward | GTGGCAAGTGTACCATGGAGA | Solyc03g115230 |
|  | Reverse | GACTTGCCTCAACTGCTCGT |  |
| *SlHsp90-1* | Forward | TGCGTTCTTGTATGGAAGTCTGC | SGN-U312354 |
|  | Reverse | TGGAC CACTTAGTCACGACCAATC |  |
| *SlHsp17.6* | Forward | ggCTgATCTTCCAgggCTTA | NM_001247296.2 |
|  | Reverse | AgCCgACTCAAgACTTTCgAT |  |
| *β-actin* | Forward | ACAgTgTCTggATTggAggC | NM_001330119.1 |
|  | Reverse | CACTACAATTgCATCTCTggTCC |  |


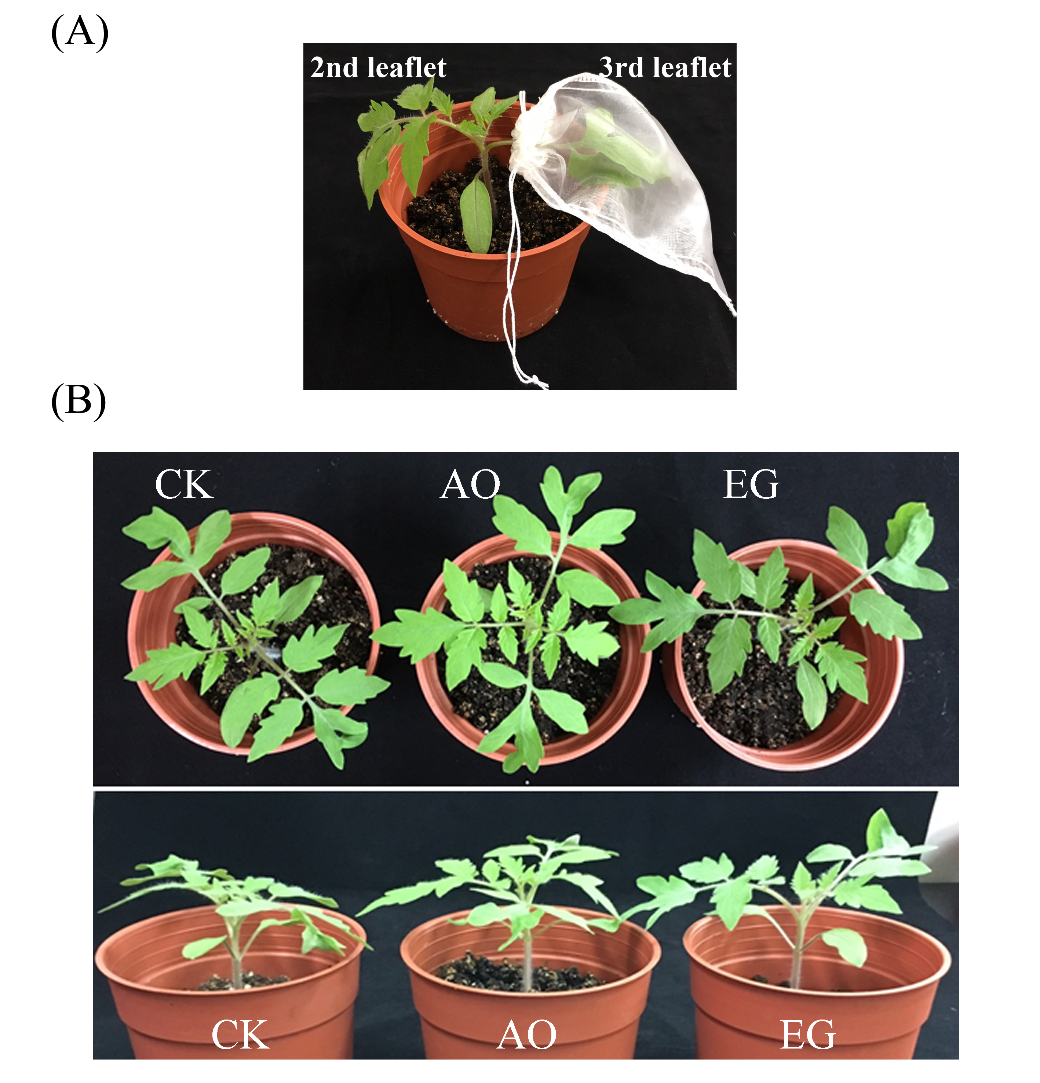


**Figure S1.** Schematic diagram of tomato leaves for TYLCTHV inoculation via viruliferous whiteflies.

(A) Seedlings with the 4-true-leaf stage were exposed to viruliferous whiteflies for TYLCTHV inoculation in order to investigate the virus infection and accumulation.

The whiteflies were enclosed in a small net bag (6 × 15 cm^2^, 110 mesh) with the 3^rd^ leaflet for a 48-h inoculation access period (IAP). Samples for gene expression analysis were harvested at the 2^nd^ leaflet from leaf apex.

(B) 12-day-old tomato seedlings were sprayed with a control solution, eugenol (200 μg mL^−1^), or anise oil (200 μg mL^−1^) three times per day. After 5 day, their phenotypes were recorded

**
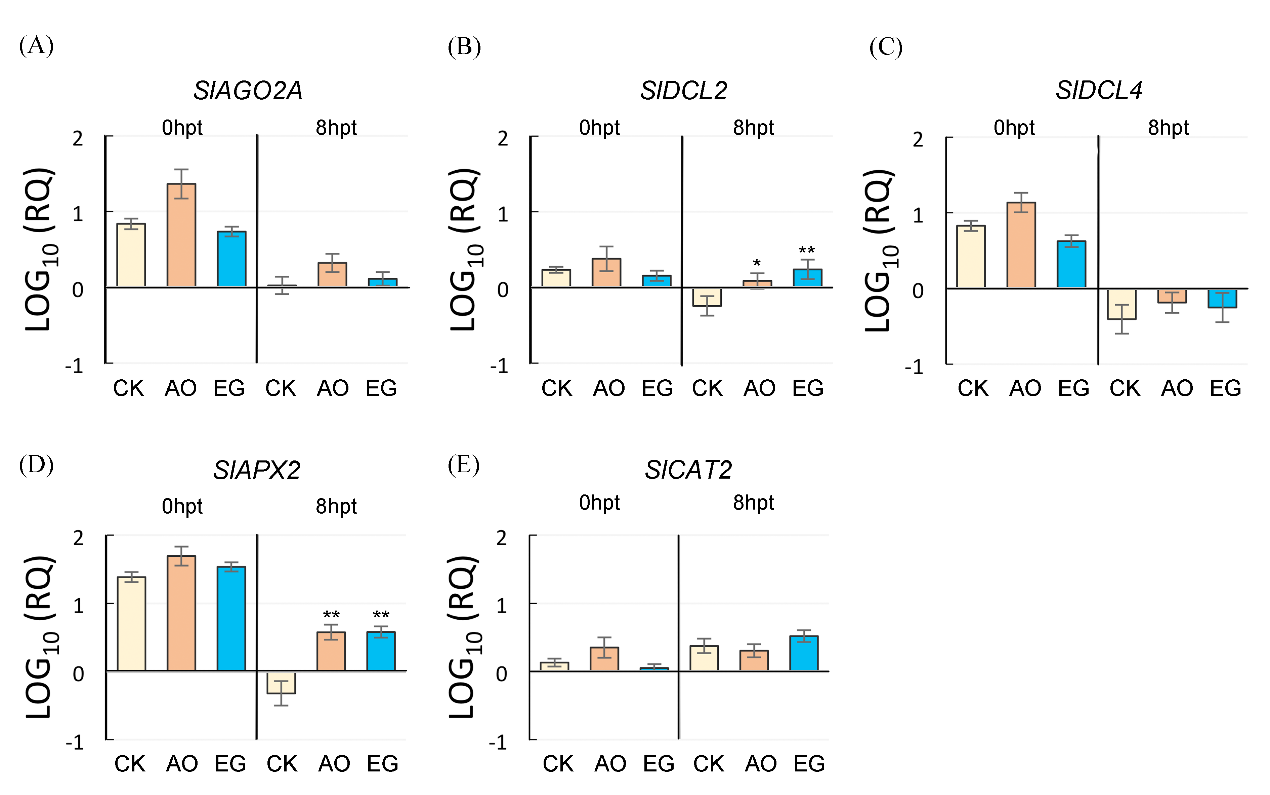
**

**Figure S2.** Effects of anise oil and eugenol on the expression of RNA silencing- and antioxidant-genes in tomato plants under heat stress.

Fourteen-day-old tomato seedlings treated with a control solution (CK), anise oil (AO), or eugenol (EG) for 24 h were subjected to heat stress at 45°C for 12 h. After heat treatment (0hpt) and 8-h recovery (8hpt), total RNA was extracted from these plants. The *SlAGO2A* (A), *SlDCL2* (B), *SlDCL4* (C), *SlAPX2* (D), and *SlXAT2* (E) expression levels were analyzed by qRT-PCR*.* The *β-actin* expression level was used as the internal control. Relative gene expression levels were normalized against the expression level in untreated tomato plants. Data were analyzed with the 2^−ΔΔCt^ method. Bars represent the mean (± standard error of the mean) of three independent biological replicates each with three technical replicates. Significant differences between control and anise oil- or eugenol-treated plants were assessed with Student’s *t*-test (*P < 0.05; **P < 0.01).


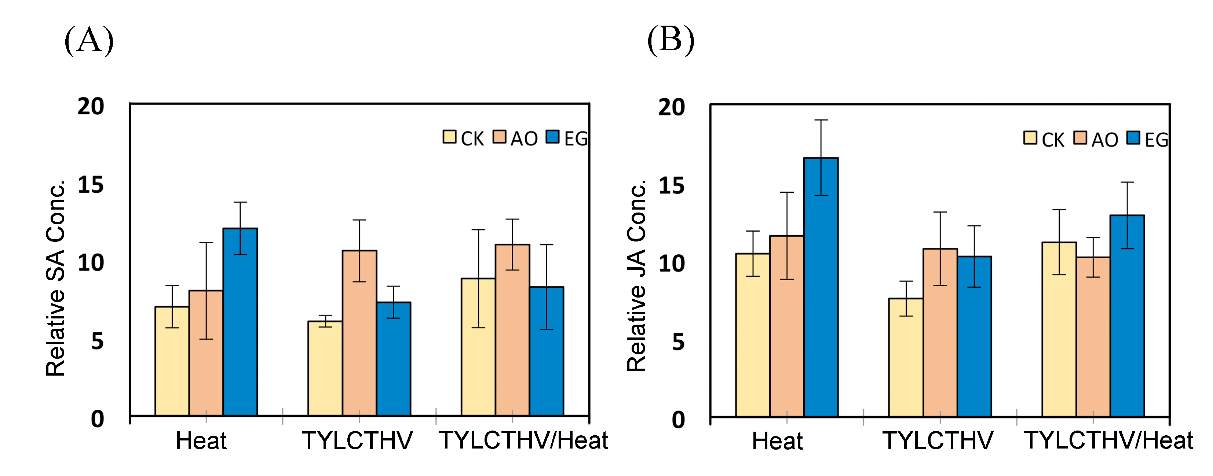


**Figure S3.** Effects of anise oil and eugenol on the endogenous salicylic acid (SA) and jasmonic acid (JA) contents under stresses.

Fourteen-day-old tomato seedlings were treated with a control solution (CK), eugenol (EG), or anise oil (AO) for 24 h were incubated at heat stress or/and inoculated TYLCTHV. The endogenous SA (A) and JA (B) contents from these plants were quantified. The SA and JA levels of the untreated tomato plants was treated as the normalized reference, with a value of one. Bars represent the mean (± standard deviation) from two measurements (each with triplicate samples). Significant differences between control and anise oil- or eugenol-treated plants were assessed with Student’s *t*-test (*P < 0.05; **P < 0.01).
